# Integrated transcriptomic and proteomic analyses of a molecular mechanism of radular teeth biomineralization in *Cryptochiton stelleri*

**DOI:** 10.1038/s41598-018-37839-2

**Published:** 2019-01-29

**Authors:** Michiko Nemoto, Dongni Ren, Steven Herrera, Songqin Pan, Takashi Tamura, Kenji Inagaki, David Kisailus

**Affiliations:** 1 Graduate School of Environmental and life Science, Okayama University, Okayama, 700-8530 Japan; 2 Materials Science and Engineering Program, University of California, Riverside, CA 92521 USA; 3 W. M. Keck Proteomics Laboratory, Institute for Integrative Genome Biology, University of California, Riverside, CA 92521 USA; 4 Department of Chemical and Environmental Engineering, University of California, Riverside, CA 92521 USA

## Abstract

Many species of chiton are known to deposit magnetite (Fe_3_O_4_) within the cusps of their heavily mineralized and ultrahard radular teeth. Recently, much attention has been paid to the ultrastructural design and superior mechanical properties of these radular teeth, providing a promising model for the development of novel abrasion resistant materials. Here, we constructed *de novo* assembled transcripts from the radular tissue of *C. stelleri* that were used for transcriptome and proteome analysis. Transcriptomic analysis revealed that the top 20 most highly expressed transcripts in the non-mineralized teeth region include the transcripts encoding ferritin, while those in the mineralized teeth region contain a high proportion of mitochondrial respiratory chain proteins. Proteomic analysis identified 22 proteins that were specifically expressed in the mineralized cusp. These specific proteins include a novel protein that we term radular teeth matrix protein1 (RTMP1), globins, peroxidasins, antioxidant enzymes and a ferroxidase protein. This study reports the first *de novo* transcriptome assembly from *C. stelleri*, providing a broad overview of radular teeth mineralization. This new transcriptomic resource and the proteomic profiles of mineralized cusp are valuable for further investigation of the molecular mechanisms of radular teeth mineralization in chitons.

## Introduction

**Figure 1:**
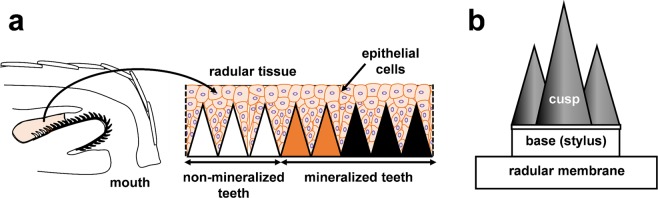
Schematics of radular tissues used in this study. (**a**) Radular tissues of the giant pacific chiton, *Cryptochiton stelleri*. (**b**) Individual radular teeth, which are composed of mineralized cusp, base and radular membrane.

Biologically formed magnetite (Fe_3_O_4_) has been found from several organisms including bacteria, chiton, homing pigeons, honeybees and salmon^1–5^. In the last few decades, significant information has been accumulated regarding bacterial magnetite formation^6–9^. In contrast, little is known about molecular mechanisms of magnetite biomineralization in eukaryotes. The magnetite-rich teeth of chitons was first reported in 1962 and its formation mechanism has been of interest for several decades^1^. Chitons have several dozen rows of teeth that are attached to a ribbon-like structure called a radular membrane. Each tooth is composed of a mineralized cusp and base (also referred to as the stylus) supporting the mineralized cusp (Fig. 1b). Magnetite is deposited only in the cusp region. Recently, ultrastructural and mechanical characterization of fully mineralized chiton teeth from *Cryptochiton stelleri* showed that it has the maximum hardness and stiffness of any known biomineral^10,11^. Although some preliminary analysis provides critical structure-mechanical property relationships in these teeth^12,13^, a further understanding of the formation machinery used to construct this ultrahard biomaterial will allow us to develop a novel abrasion resistant material.

We have studied magnetite biomineralization in the world’s largest species of chiton, *C. stelleri*. As other chitons, *C. stelleri* use their teeth to scrape the algae growing on intertidal rocks. When these teeth become worn, they are replaced by a new row of teeth that are continuously formed from inside a radular sac^14^. Therefore, it is possible to observe all stages of tooth development within one radula. In the radular teeth of *C. stelleri*, no mineral deposits are found on the first 8–12 rows of teeth. These non-mineralized teeth are transparent and are mainly composed of α-chitin and proteins. The next 2–5 rows of teeth turn a reddish-brown color, representing a partial mineralization of the teeth with an amorphous or weakly nanocrystalline iron oxide (ferrihydrite). The subsequent rows of teeth have black-colored cusps, indicative of a transformation from ferrihydrite to magnetite towards fully mineralized teeth. Detailed observations of developing teeth revealed that the mineral aggregates of ferrihydrite first deposited on alpha-chitin fibers continuously grow in size as teeth maturation proceeds. Phase transformation to magnetite occurred within a few rows of teeth after the onset of ferrihydrite formation. Although radular membrane, base and cusp are all composed of alpha-chitin, iron-based mineral was deposited only in the cusp region. From these results, we assume that there may be several proteins that dictate specific iron oxide nucleation in the cusp region as well as transformation to magnetite in chiton teeth^15,16^. To reveal the proteins involved in the formation of radular teeth, we previously conducted proteome analysis of mineralized teeth of *C. stelleri* using a *de novo* peptide sequencing technology^15^. However, because of the lack of genomic sequence information, only partial sequences of mineralized teeth proteins were obtained.

In this study, to better understand the molecular mechanisms of magnetite biomineralization, we use RNA sequencing (RNA-seq) to construct the transcriptome data set of radular tissue in *C. stelleri*. RNA-seq based transcriptome analysis reveals highly expressed transcripts in both non-mineralized and mineralized teeth regions. Furthermore, the transcriptome data was used to identify the proteins in mineralized teeth.

## Results

### RNA-seq and *de novo* transcriptome assembly

Radular teeth are formed within the radular tissue. Here, teeth are surrounded by epithelial cells (Fig. 1a) and it is likely that the proteins required for teeth formation may be supplied from these cells. To construct expressed sequence data sets from the radular tissue, RNA was extracted from this tissue (including radular teeth and epithelial cells). After dissection, radular tissue from the non-mineralized teeth region (first 8 to 12 rows of transparent teeth) and those from the mineralized teeth region (following 2 to 5 rows of partially-mineralized reddish-brown colored teeth plus 5 to 6 rows of mineralized magnetic teeth) (Fig. 1a) were separated and RNA was extracted from each sample. RNA-seq was conducted on the extracted RNA. The reads obtained from the non-mineralized teeth region and the mineralized teeth region were then assembled together into 187,980 transcripts. The average length of transcripts is 705 bp and the maximum length is 16,738 bp (with a N50 length of 1,110 bp, Table 1).

**Table 1:** Summary of *C. stelleri* RNA-seq results.

Sequencing summary	
Number of reads	
non-mineralized teeth region	43,802,270
mineralized teeth region	45,141,430
**Assembly summary**	
Number of transcripts	187,980
Average length of transcripts (bp)	705
N50 length (bp)	1,110
Maximum length of transcript (bp)	16,738
**Mapping summary**	
Total mapped reads	
non-mineralized teeth	35,343,878
mineralized teeth	35,511,782
% of Total mapped reads	
non-mineralized teeth (%)	80.69
mineralized teeth (%)	78.67

### Highly expressed transcripts in mineralized and non-mineralized teeth regions

To detect highly expressed transcripts in mineralized and non-mineralized teeth region, each set of reads from those samples was first aligned to the assembled 187,980 transcripts.

Among the twenty most highly expressed transcripts in the non-mineralized teeth region, five transcripts are isoforms of Cs17717|c0_g1, which did not show similarity to any known proteins (Supplementary Table S1). There are two transcripts encoding peritrophin-like proteins: Cs79475|c0_g1_i1 and Cs70642|c0_g2_i1. Peritrophin and peritrophin-like proteins have been found in both insects and crustaceans. These proteins have chitin binding domains and have been thought to be involved in immune defense or food digestion^17^. Cs25220|c1_g1_i1 shares a similarity to a calcium carbonate biomineralization related protein, Pif, in mollusks^18^. Four proteins, including peritrophin-like and Pif-like proteins, described above have a type-2 chitin-binding domain^19^. The transcripts of a cytochrome c oxidase subunit I (Cs76629|c0_g1_i1) and a ferritin subunit 2 (Cs75144|c0_g1_i1) that are involved in iron accumulation were included in highly expressed transcripts in the non-mineralized teeth region. Furthermore, four transcripts encoding radular teeth proteins, which are mentioned below, were also included.

Distinct profiles were observed in the twenty most highly expressed transcripts in the mineralized teeth region (Supplementary Table S2). The mitochondrial respiratory chain enzymes constitute 35% of the highly expressed transcripts. Those enzymes include cytochrome c oxidase subunit I, cytochrome c oxidase subunit II, cytochrome c oxidase subunit III, ATP synthase F0 subunit 6, NADH dehydrogenase subunit 5 and NADH dehydrogenase subunit 1. Those are encoded in mitochondrial genome of *C. stelleri*^20^. In addition, the chitin binding beak protein 3 (Cs12250|c0_g1_i1), which has been found from squid beaks was included. Five transcripts (25%) are isoforms of Cs22243|c0_g7, whose function is unknown.

### Analysis of ferritin genes

Ferritin is an iron transport and storage protein which assembles to form a protein cage containing up to 4500 iron atoms in the form of a hydrous ferric oxide-based mineral. Since ferritins were detected and isolated from epithelial cells of the radular tissue in *C. stelleri*^21^, ferritin has been thought to be involved in iron transport for tooth mineralization. In this study, the transcripts showing homology with ferritin genes were screened from the 134,993 transcripts. As a result, the deduced amino acid sequences of the four transcripts share homology with ferritin protein from the chiton *Liolophura japonica*. Among four ferritin homologs, two are identical except one amino acid substitution (Cs22563|c0_g1_i1 and Cs75144|c0_g1_i1) (Supplementary Fig. S1). The sequence of Cs17042|c0_g1_i1 lacked the 5′ terminus of the full-length cDNA. Until now, two types of ferritins, cytoplasmic ferritins and secreted ferritins, have been isolated from mollusks^22^. The results of a homology search indicated that Cs90734|c0_g1_i1 shared a homology with secreted ferritins, while Cs22563|c0_g1_i1, Cs75144|c0_g1_i1 and Cs17042|c0_g1_i1 are more similar to cytoplasmic ferritins. The phylogenetic analysis was conducted using the sequences of known secreted and cytoplasmic ferritins from Mollusca and Arthropoda. As a result, Cs90734|c0_g1_i1 ferritin clustered with secreted ferritins and the other three ferritins clustered with cytoplasmic ferritins (Supplementary Fig. S2). Evidently, compared to other ferritins in *C. stelleri*, Cs90734|c0_g1_i1 has distinct structural features commonly observed in secreted-type ferritins such as: (1) a ~40 amino acid residue insertion located in the middle of the putative loop region, (2) the putative metal binding residues, which are highly conserved in cytoplasmic ferritins, are replaced with other amino acids. However, it does not contain signal sequences that exist in other secreted ferritins. Among four ferritin genes, Cs75144|c0_g1_i1 showed a predominant expression, suggesting its importance in radular teeth formation (Supplementary Table S3). Notably, Cs75144|c0_g1_i1 was more highly expressed in the non-mineralized teeth region than in the mineralized teeth region.

### Proteome analysis of radular teeth proteins

In chitons, each radular tooth consists of a mineralized cusp, base (also referred to as stylus) supporting the mineralized cusp and a radular membrane (Fig. 1b). Previously, the mineralized cusps were physically separated from base plus radular membrane and the proteins were extracted from each part of radular teeth using boiling SDS^15^. Extracted proteins were digested into peptides and analyzed with nano-LC-MS. In that work, the lack of available genetic information on *C. stelleri* required a MS/MS ion search against the NCBInr database and *de novo* peptide sequencing of MS/MS spectra in order to identify the proteins.

In this study, a full six-frame translated database was initially generated from assembled transcripts of *C. stelleri*. Then, the previously obtained MS/MS spectra of teeth proteins were analyzed by a MS/MS ion search using the translated database. Data sets of MS/MS spectra from replicate analyses (n = 2) were used for the search. As a result, the peptide sequences from the mineralized cusp fraction were assigned to 232 transcripts (Supplementary Table S4) and those from the base plus membrane fraction were assigned to 114 transcripts (Supplementary Table S5). A high level of redundancy was present in the results as many of the peptides were shared by different proteins. This could be due to the presence of isoforms or artificial redundancy caused by sequencing errors or assembly errors^23^. The proteins matching the same set of peptides are listed in Supplementary Tables S4 and S5. From FPKM expression data, the proteins encoded by the transcripts which showed the greatest expression were selected for further analysis. Annotations of the proteins are based on the results of BlastP analyses.

In total, 31 proteins were identified from the base plus membrane fraction (Supplementary Table S5). From this, 22 proteins were identified from 1^st^ analysis and 24 proteins were identified from 2^nd^ analysis. From the cusp fraction, a total of 77 proteins were identified. 61 proteins were identified from the 1^st^ analysis and 54 proteins were identified from the 2^nd^ analysis. The proteins identified from the cusp fraction include four proteins: Myoglobin, actin, elongation factor 1 alpha and Arginine kinase, which are encoded by the top 20 most highly expressed transcripts in the non-mineralized teeth region (Cs77196|c0_g2_i1, Cs47470|c2_g3_i1, Cs24354|c0_g1_i1 and Cs82664|c0_g1_i1; Supplementary Table S1). An iron transporting protein, ferritin, whose gene was also highly expressed in non-mineralized teeth region, was not identified. The 19 proteins were identified from both cusp and base plus membrane fractions. It includes a large number of structural proteins such as actin, filamin-A, tubulin and fibrillin. Elongation factor 1 alpha (Cs24354|c0_g1_i1) was identified from a blank sample and is thought to be a contaminant.

### Mineralized cusp-specific proteins

The mineralized cusp proteins, which were identified in both 1^st^ and 2^nd^ analyses (and not identified from the base plus membrane fraction), were listed as mineralized cusp-specific proteins (Table 2).

**Table 2:** List of mineralized cusp-specific proteins in *C. stelleri*.

Transcript ID	Annotation^a^	Best hit organisms	E-value	FPKM (mineralized)	FPKM (non mineralized)	1st analysis		2nd analysis	
						Mascot scores	Peptides	Mascot scores	Peptides
Cs77024|c0_g1_i1	Neuroglobin-like	*Actinia tenebrosa*	2.00E-25	2323.65	2020.22	845	4	428	4
Cs77196|c0_g2_i1	Myoglobin	*Liolophura japonica*	3.00E-84	874.48	5345.03	387	4	234	3
Cs75674|c0_g4_i1	protein usf-like	*Octopus bimaculoides*	1.00E-101	67.74	2144.29	331	5	54	1
Cs82664|c0_g1_i1	Arginine kinase	*Liolophura japonica*	0	104.69	2469.32	189	4	178	2
Cs18149|c2_g1_i5	Peroxidasin	*Crassostrea gigas*	0	12.02	0.18	173	3	195	3
Cs68435|c0_g1_i1	No hits found			309.95	75	126	2	88	1
Cs83384|c0_g1_i1	protein yellow-like	*Lingula anatina*	3.00E-103	18.69	2.05	109	1	90	1
Cs18149|c2_g1_i2	Peroxidasin	*Mizuhopecten yessoensis*	1.00E-19	1.98	0	102	2	111	3
Cs62719|c1_g8_i1	gastric intrinsic factor-like protein 2	*Hyriopsis cumingii*	2.00E-35	28.05	0.08	114	1	56	1
Cs58153|c0_g2_i5	apoptosis inducing factor-3	*Haliotis discus discus*	0.00E + 00	62.87	16.52	95	1	98	2
Cs22616|c0_g1_i1	copper transport protein ATOX1-like	*Exaiptasia pallida*	3.00E-27	2324.41	144.53	109	1	104	1
Cs28725|c1_g1_i1	hillarin	*Crassostrea virginica*	0	15.52	62.53	83	1	61	1
Cs84529|c0_g2_i1	muscle-specific protein 20-like isoform X2	*Lingula anatina*	7.00E-91	999.04	345.27	97	1	74	1
Cs59400|c0_g1_i1	Eukaryotic initiation factor 4A-II, partial	*Stegodyphus mimosarum*	0	145.13	141.93	95	1	85	1
Cs74134|c0_g2_i2	histone H2B 8-like	*Protobothrops mucrosquamatus*	2.00E-68	6.85	51.47	91	1	127	1
Cs46312|c2_g1_i1	hephaestin-like protein	*Stylophora pistillata*	0	0.27	179.74	90	1	84	2
Cs54305|c0_g1_i2	filamin-A-like isoform X1	*Biomphalaria glabrata*	0	125.51	91.37	75	1	147	2
Cs59318|c0_g1_i1	peroxiredoxin 6	*Cristaria plicata*	4.00E-119	274.95	561.11	77	2	93	2
Cs71263|c1_g1_i1	Peroxidasin	*Crassostrea gigas*	0	9.37	0.08	55	1	70	1
Cs69741|c0_g1_i1	superoxide dismutase (Cu-Zn)	*Drosophila sechellia*	6.00E-46	46.09	22.35	76	1	190	2
Cs53984|c1_g2_i8	filamin-A-like isoform X5	*Crassostrea virginica*	2.00E-104	10.13	4.09	69	1	132	2
Cs28687|c2_g1_i1	cytosolic malate dehydrogenase	*Mytilus galloprovincialis*	3.00E-176	34.88	81.1	61	1	59	1

^a^Annotation is based on the results of BlastP analysis.

Two globin-like proteins were identified as mineralized cusp-specific proteins with the highest MASCOT scores (Cs77196|c0_g2_i1 and Cs77024|c0_g1_i1). The sequences of both proteins contain heme-binding domains of globin proteins. Cs77196|c0_g2_i1 showed the best match with radular muscle myoglobin of *Liolophura japonica*^24^. This protein is abundant in the cusp region and its N-terminal sequence was determined in our previous study^15^. Cs77024|c0_g1_i1 showed a similarity with a neuroglobin-like protein. An acidic peptide sequence detected in the previous study which was not assigned to any protein at that time was the part of Cs77024|c0_g1_i1^15^. Sequence alignment with other neuroglobins revealed that the acidic region of Cs77024|c0_g1_i1 was unique to this protein.

Three proteins shared a similarity with peroxidasin. Among them, two were isoforms: Cs18149|c2_g1_i5 and Cs18149|c2_g1_i2. All peroxidasin possess N-terminal signal sequences and heme-dependent peroxidase domains (Supplementary Table S6). Furthermore, a chitin-binding domain was found at the C-terminus of Cs18149|c2_g1_i5. A number of peroxidasin homologues are in the transcriptome data, and although there are 3′- or 5′- truncated forms, only Cs18149|c2_g1_i5 has a chitin-binding domain.

Two proteins correspond to antioxidant enzymes, peroxiredoxin 6 (Cs59318|c0_g1_i1) and superoxide dismutase (Cu-Zn) (Cs69741|c0_g1_i1). As a result of subcellular localization analysis, peroxiredoxin 6 was predicted as a cytoplasmic protein, while superoxide dismutase (Cu-Zn) was predicted as an extracellular protein (Supplementary Table S6).

Two proteins were homologous to the metal transport proteins, such as a copper transport protein ATOX1-like (Cs22616|c0_g1_i1) and a hephaestin-like protein (Cs46312|c2_g1_i1). Hephaestin is a protein that possesses ferroxidase activity and is believed to mediate intestinal iron absorption in mammals^25,26^. Both membrane-associated and soluble forms of hephaestin have been reported^27,28^. A domain search predicted the presence of a chitin-binding domain in hephaestin-like protein (Cs46312|c2_g1_i1). It has three predicted β-sheet structures and several aromatic amino acids that are characteristic to a type-3 chitin-binding domain^29^. Cs22616|c0_g1_i1 has a heavy metal-associated domain of ATOX1, which contain two cysteine residues for metal ion binding. ATOX1 is a copper chaperone that plays a role in incorporating Cu into the copper-dependent enzymes such as hephaestin^30^.

Other proteins include Arginine kinase (Cs82664|c0_g1_i1), hillarin (Cs28725|c1_g1_i1) and cytosolic malate dehydrogenase (Cs28687|c2_g1_i1), which are predicted to localize in the mitochondria (Supplementary Table S6). Furthermore, nuclear proteins (Eukaryotic initiation factor 4A-II, partial and histone H2B 8-like) and cytoskeletal proteins (muscle-specific protein 20-like isoform X2 and filamin-A-like isoforms) were identified. Protein usf-like (Cs75674|c0_g4_i1) that has a dienelactone hydrolase domain was identified with a high MASCOT score and a 33% sequence coverage.

**Figure 2:**
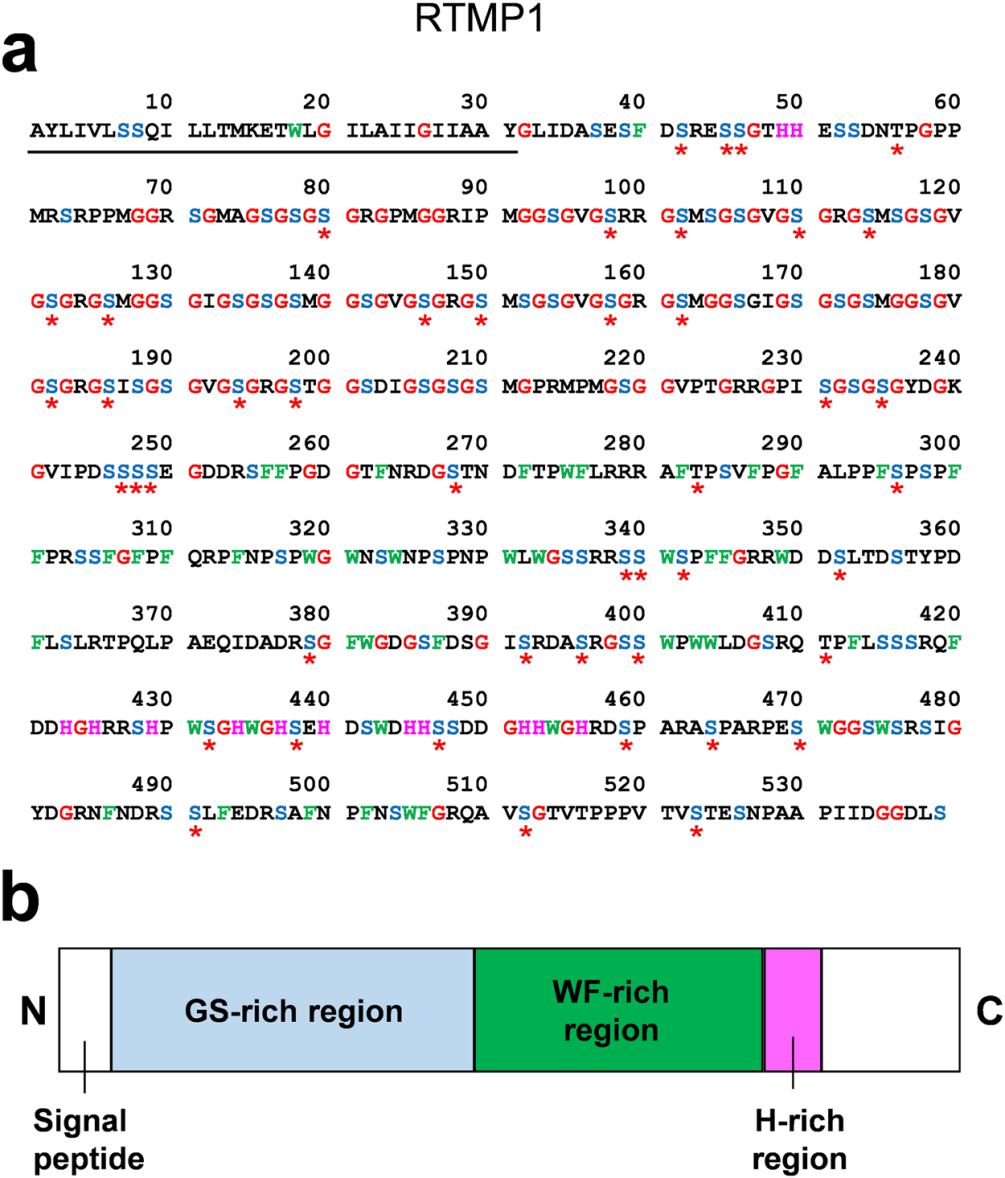
Amino acid sequence of a novel mineralized cusp-specific protein, radular teeth matrix protein 1 (RTMP1). (**a**) The entire amino acid sequence of RTMP1 deduced from the cDNA sequence. It consists of 539 amino acids and abundant in glycine (red) and serine (cyan). The underline indicates a putative signal sequence. The putative phosphorylation sites are indicated by asterisks. (**b**) Schematic of the domain structure of RTMP1. GS-rich repetitive region, WF-rich, and H-rich regions are represented by pale blue, green, and pink boxes.

There is only one protein (Cs68435|c0_g1_i1) that shared no sequence similarity with any known proteins. This protein possesses a unique repetitive sequence and an unusually high proportion of glycine and serine residues. Further analysis was conducted on this novel protein. Since the 5′ terminus of the Cs68435|c0_g1_i1 coding sequence was truncated, its full-length sequence was determined by 5′-RACE. The overall sequence consists of a glycine- and serine-rich repetitive region, followed by a tryptophan- and phenylalanine-rich region and a histidine-rich region (Fig. 2). Structure prediction indicates that a disordered structure is present in a glycine- and serine-rich region (Supplementary Fig. S4). A sequence alignment of a WF-rich region in RTMP1 with the chitin-binding domains of four bacterial chitinases indicated that three of five putative chitin interacting residues in the chitin-binding domains were also conserved in RTMP1 (Supplementary Fig. S5)^29^. Three β-strands were predicted in the corresponding region of RTMP1. Based on results of *in silico* analyses, a number of serine residues of Cs68435|c0_g1_i1 are predicted to be phosphorylated. Analyses by several different prediction software indicated that Cs68435|c0_g1_i1 contains an N-terminal signal sequence (Fig. 2, Supplementary Table S6). This novel protein is termed RTMP1 (radular teeth matrix protein 1).

### Gene expression profiles of mineralized cusp-specific proteins

The expression profiles of the transcripts encoding mineralized cusp-specific proteins in different mineralization stages were analyzed based on FPKM values. Expression levels of several transcripts (e.g., Neuroglobin-like, peroxiredoxin 6 and superoxide dismutase (Cu-Zn)) differed in abundance by less than 4-fold between the mineralized teeth and non-mineralized teeth regions (Supplementary Fig. S3). On the other hand, the transcripts whose expression increased more than 4-fold in the non-mineralized teeth region (e.g., protein usf-like, Arginine kinase, Myoglobin and hephaestin-like protein) or mineralized teeth region (e.g., RTMP1, Peroxidasin and copper transport protein ATOX1-like) are thought to be differentially expressed during teeth mineralization (Supplementary Fig. S3).

## Discussion

In this study, a comprehensive transcriptome data from the radular tissue of *C. stelleri* has been developed for the first time. The constructed transcriptome data provides new insights into the genes highly expressed in radular tissue during teeth formation in *C. stelleri*. Furthermore, by using the transcriptome data, a proteome profile of mineralized cusp-specific proteins were determined. Those transcriptome and proteome data represent invaluable resources for the investigation of the magnetite biomineralization (including iron oxide nucleation, growth and phase transformation) in chitons.

Four homologues of ferritin were identified from the transcriptome data. Among the four ferritin homologs, the cytoplasmic ferritin, Cs75144|c0_g1_i1 is highly expressed in radular tissue. Previously, it was demonstrated that ferritin-containing granules first appear in the epithelial cells located 1–3 rows anterior to the tooth in which iron oxide is first deposited. The amount of granules increases greatly over a few rows, and then gradually decreases^14^. Those observations were correlated with the expression pattern of cytoplasmic ferritins (Supplementary Table S3). Since the iron transport is the earliest event in the tooth formation process, the expression of the ferritin genes might be strongly induced in the non-mineralized teeth region prior to mineralization.

Contrary to the above data, previous research using other chiton species suggested that secreted ferritins accumulated in the epithelial cells of radular tissue during teeth mineralization^14,31^. In order to determine which ferritin plays a role in teeth mineralization, further studies including protein analysis of epithelial cells are needed.

Although the ferritin gene, Cs75144|c0_g1_i1 was included in the twenty most highly expressed transcripts in the non-mineralized teeth region (Supplementary Table S1), ferritin proteins were not identified from mineralized cusps. This result was consistent with the previous observation that no ferritin granules were detected inside the teeth^32^. Based on these results, it is likely that iron in ferritin is released and transported inside the teeth in a soluble form, and re-deposited as ferrihydrite. Shaw *et al*. reported the existence of ferritin like granules in the cells located inside of the base (also referred to as stylus)^33^. However, in this study, we did not detect ferritin from the base plus membrane fraction (Supplementary Table S5). It is possible that sample preparation of the base plus membrane fraction that included repeated wash with 70% ethanol and water^15^, could result in the loss of ferritin proteins.

The mitochondrial respiratory chain enzymes were overrepresented among the top-20 most highly expressed transcripts in the mineralized teeth region (Supplementary Table S2). Electron microscopic analysis of partially mineralized teeth have previously shown the presence of numerous mitochondria in the cells surrounding the partially mineralized teeth^14,34^. It has been suggested that a large amount of energy required for iron transport or transformation of ferrihydrite to magnetite may be provided by those mitochondria^14,34^.

Proteomic analysis using the *de novo* constructed transcriptome data of radular tissue in *C. stelleri* led to the identification of three-fold more mineralized cusp-specific proteins (22 in total) than our previous report^15^. Furthermore, a novel protein that could not be detected by the previous method was identified in this study.

Cytoskeletal proteins (e.g., filamin-A-like isoforms, muscle-specific protein 20-like isoform X2) or nuclear proteins (e.g., Eukaryotic initiation factor 4A-II, partial, histone H2B 8-like), which were apparently unrelated to teeth formation, were identified as mineralized cusp-specific proteins (Table 2). It is likely that those highly expressed proteins were occluded in the teeth during mineralization and identified as mineralized cusp proteins.

Two globin-like proteins were identified as mineralized cusp-specific proteins. Significant amounts of myoglobin, which help supply oxygen to muscle^35^, have been known to exist in the radular muscles of chiton^24,36,37^. Therefore, it is likely that the highly expressed myoglobin could contaminate the mineralized cusp fraction. Conversely, as the oxygen concentration strongly affects the redox state of iron, iron release from ferritin and magnetite formation, those proteins could have essential roles for teeth formation and mineral phase transformation. Although the function of the neuroglobin-like protein, Cs77024|c0_g1_i1, is unknown, it has been suggested to play a role in O_2_ control as myoglobin^38^. Of note, the neuroglobin-like protein, Cs77024|c0_g1_i1, has a unique acidic region that was not present in other neuroglobin-like proteins. The acidic domain of the magnetite morphology-modulating protein, Mms6 has been shown to be essential for its function^39^. Therefore, Cs77024|c0_g1_i1, which has a unique acidic amino acid rich region, might have a specialized function for magnetite formation.

3 out of 22 mineralized cusp-specific proteins (Cs18149|c2_g1_i5, Cs18149|c2_g1_i2 and Cs71263|c1_g1_i1) showed similarities to peroxidasin. Peroxidasin is known to have a hybrid structure consisting of a peroxidase domain and extracellular matrix motifs^40^. It is secreted extracellularly and mediates the cross-link formation in collagen for tissue development^41^. Notably, among all peroxidasin-like proteins in transcriptome data, only Cs18149|c2_g1_i5 has a chitin binding domain in its C-terminus. It is possible that Cs18149|c2_g1_i5 is secreted extracellularly to bind chitin and catalyze the chemical bond formation to consolidate a scaffold for biomineralization. Interestingly, peroxidasin has been identified from other calcium-based biominerals, including pearl oyster, and has been suggested to be important for biomineral formation^42–44^.

The biomineralization protein Silicatein, which was isolated from sponge spicules, has been known to catalyze the hydrolysis and polycondensation of silicon alkoxide to form silica^45,46^. We speculate that the usf-like protein (Cs75674|c0_g4_i1), containing a dienelactone hydrolase domain, could also have a catalytic role contributing to hydrolysis of iron oxide precursors to form magnetite^47^.

Two antioxidant proteins, peroxiredoxin 6 (Cs59318|c0_g1_i1) and superoxide dismutase (Cu-Zn) (Cs69741|c0_g1_i1) were predicted to be localized both inside and outside of the cells (Supplementary Table S6). Since the proposed models of iron release pathways from ferritins involved superoxide radicals^48^, superoxide dismutase (Cu-Zn) (Cs69741|c0_g1_i1) could control excess amounts of superoxide radicals. As we mentioned in our previous work, peroxiredoxin 6 (Cs59318|c0_g1_i1) may function to control the reduction of iron species and therefore control the initial phase transformation from ferrihydrite to magnetite by adsorption of Fe^2+^ ions with subsequent solid-state transformation^16^.

A hephaestin-like protein (Cs46312|c2_g1_i1) is a ferroxidase protein and may be involved in ferrous iron oxidation into ferric iron. Of note, hephaestin-like protein contains an N-terminal signal sequence (Supplementary Table S6) and a chitin-binding domain. It suggests that extracellularly secreted hephaestin-like proteins may bind with chitin and catalyze ferrous iron oxidation.

A novel mineralized cusp-specific protein, RTMP1, has a distinctive glycine and serine-rich region (Fig. 2). Similar glycine- and serine-rich domains were found from other biomineral-associated proteins^49–51^. The secondary structure of a glycine and serine-rich region was predicted to be disordered and thus might form a flexible structure like the aforementioned biomineral proteins (Supplementary Fig. S4). It is possible that the extended conformation of RTMP1 might provide more mineral binding sites than tightly packed globular proteins^52^. The WF-rich region in RTMP1 resembles a type-3 chitin binding domain of bacterial chitinase, which have β-sheets as well as aromatic and hydrophobic-rich sequences (Supplementary Fig. S5). Thus, similar to those bacterial chitinases, it is likely that RTMP1 binds to crystalline forms of chitin^29^. A number of phosphorylated serines were inferred to reside in RTMP1. Of note, amino acid analysis of matrix proteins in the radula teeth of the chiton *Acanthopleura hirtosa* indicated that there were appreciable amounts of phosphorylated serines^53^. In other biominerals, phosphorylation is often required for mineral-protein interaction^54,55^. These results support the idea that the extracellularly secreted RTMP1, which is likely to be phosphorylated, may interact with chitin and promote iron oxide nucleation on chitin fibers.

Expression analysis of the transcripts encoding the mineralized cusp-specific proteins provides additional insight into the functions of those proteins for teeth mineralization. Except for the aforementioned possible contaminants, the transcripts highly expressed throughout teeth mineralization (e.g., Neuroglobin-like, peroxiredoxin 6 and superoxide dismutase (Cu-Zn)) may be important for the entire process of mineralization (Supplementary Fig. S3). Conversely, the transcripts highly expressed in the non-mineralized teeth region (e.g., protein usf-like, Arginine kinase, hephaestin-like protein) may play a role in the former processes, including initial iron transport (Supplementary Fig. S3). The transcripts highly expressed in the mineralized teeth region (e.g., RTMP1, Peroxidasins, copper transport protein ATOX1-like) may be involved in latter processes, such as ferrihydrite nucleation, growth, and phase transformation to magnetite (Supplementary Fig. S3).

By analyzing gene deletion mutants, several proteins important for magnetite mineralization in magnetotactic bacteria have been reported^6–9^. Those proteins include redox-active proteins that may control oxidation and reduction of iron for magnetite mineralization^56,57^, magnetotactic bacteria-specific proteins related to maturation of magnetite crystals^6,7^, cation diffusion facilitators^58^ and proteins with protease domains^59^. When compared to the magnetotactic bacterial proteins, redox-active proteins (peroxiredoxin-6, superoxide dismutase (Cu-Zn) and hephaestin-like protein) were also identified from *C. stelleri* as mineralized cusp-specific proteins. These proteins may play roles in oxidation and reduction of iron for transport, nucleation and magnetite mineralization.

## Conclusions

In this study, we constructed the first comprehensive transcriptome data sets from radular tissue in the gumboot chiton, *Cryptochiton stelleri*. Transcript expression analysis revealed that the most expressed transcripts in the non-mineralized teeth region include ferritin genes, while those in the mineralized teeth region include genes of a number of mitochondrial respiratory chain enzymes. These results concur with the previous microscopic observation of developing radular teeth in chitons and suggest the importance of these genes in teeth mineralization. Furthermore, using proteomic analysis, 22 mineralized cusp specific proteins including a novel protein were identified. These proteins are good candidates for future research of iron oxide mineralization in chitons. The results obtained from this study are valuable in terms of providing the first overview of radular teeth formation.

## Methods

### RNA isolation and cDNA library construction

Live specimens of *C. stelleri* were collected and maintained as previously described^16^. Collection of *Cryptochiton stelleri* is permitted by the Department of Fish and Game and the California Natural Resources Agency. Radular tissues were isolated from freshly dissected *C. stelleri*. After radular tissue isolation, non-mineralized teeth region (first 8 to 12 rows of transparent teeth) and mineralized teeth region (following 2 to 5 rows of partially-mineralized reddish-brown colored teeth plus 5 to 6 rows of mineralized magnetic teeth) were separated from whole radulae and frozen immediately in liquid nitrogen and stored at −80 °C until used. Total RNA was extracted from each radular tissue region separately using TRI reagent (Molecular Research Center), and purified using RNeasy mini kit (QIAGEN) following the manufacturer’s instructions. Integrity and quantity of extracted RNA was assessed using Agilent 2100 Bioanalyzer (Agilent Technologies). cDNA Libraries were prepared using the TruSeq Stranded mRNA Sample Prep Kit (Illumina).

### Sequencing and *de novo* transcriptome assembly

The constructed libraries were sequenced on an Illumina Hiseq 2500 platform and paired-end reads were generated. From raw reads, adapter sequences were removed using cutadapt^60^. Reads were then quality trimmed using Trimmomatic^61^. The trimmed reads from two samples (non-mineralized teeth region and mineralized teeth region) were pooled and assembled *de novo* into a transcriptome reference using Trinity^62^.

### Bioinformatic analysis

The trimmed reads obtained from two samples (non-mineralized teeth region and mineralized teeth region) were aligned to the above constructed transcriptome reference using Bowtie^63^. Based on the mapping results, the expression levels of transcripts were estimated by fragments per kilobase of transcript per million fragments mapped value (FPKM) using RSEM^64^. A homology search was conducted using the BLAST program^65^ against NCBI nr database for functional annotation of transcripts and proteins. Homologues of ferritin genes were searched from the transcriptome data of *C. stelleri* using local BLAST. The conserved domains of protein sequences were searched with SMART^66^. MEGA version 6^67^ was used for phylogenetic analysis of ferritin proteins with the maximum likelihood (ML) method. The following ferritin proteins from Mollusca and Arthropoda were used: LjFer (*Liolophura japonica*, BAA21810), MmxFer (*Meretrix meretrix*, AAZ20754), ScFer (*Sinonovacula constricta*, ACZ65230), AiFer2 (*Argopecten irradians*, AEN71561), AiFer3(*Argopecten irradians*, AEN71564), PyFer1 (*Patinopecten yessoensis*, AGK92812), PyFer2 (*Patinopecten yessoensis*, AGK92813), PyFer4 (*Patinopecten yessoensis*, AGK92815), LsFer (*Lymnaea stagnalis*, CAA40097), CrFer-H1 (*Carcinoscorpius rotundicauda*, AAW22505), CrFer-H2 (*Carcinoscorpius rotundicauda*, AAW22506), HddFer (*Haliotis discus discus*, ABG88845), EsFer1 (*Eriocheir sinensis*, ADF87490), EsFer2 (*Eriocheir sinensis*, ADF87491), PyFerS1 (*Patinopecten yessoensis*, AHH31562), PyFerS2 (*Patinopecten yessoensis*, AHH31563), SdFer (*Suberites domuncula*, CAC84556). The analysis was run with the Jones-Taylor-Thornton (JTT) model. Bootstrap trials were replicated 1000 times. The putative phosphorylation sites of RTMP1 was predicted in silico using NetPhos 3.1 Server (http://www.cbs.dtu.dk/services/NetPhos/)^68^. The potential phosphorylation sites with scores higher than 0.9 were considered to be positive. Disordered regions in RTMP1 were predicted using the DISOPRED server^69^. Jpred^70^ was used to predict the secondary structures of the WF-rich region in RTMP1. Subcellular localization of identified proteins were predicted using the following software: SignalP 4.1^71^, TargetP1.1^72^, WOLF PSORT^73^, MultiLoc2^74^ and Signal-BLAST^75^. Amino acid sequence alignments were conducted using Clustal Omega software^76^.

### Proteomic analysis

A detailed method of protein extraction from the radular teeth of *C. stelleri* and following LC-MS/MS analysis were described in our previous work^15^. In this study, first, a six frame translated database was created from a transcriptome data of *C. stelleri*. Then the previously obtained MS/MS spectra of radular teeth proteins were searched against a six-frame translated database with the MASCOT algorithm for protein identification. The parameters used for MASCOT were same as previously described^15^. The protein identification was based on two technical replicates.

### 5′ Rapid amplification of cDNA ends (RACE)

5′ rapid amplification of cDNA ends (RACE) was performed with 5′-Full RACE Core Set (Takara) to determine the full length sequences of Cs68435|c0_g1_i1 and Cs59318|c0_g1_i1. Total RNA was extracted from radular tissue as described above. 5′-RACE products were cloned into pMD20-T vector (Takara) and sequenced.

## Supplementary information

Supplementary Information

## Data Availability

The reads derived from Illumina Hiseq of the analyzed samples have been deposited in DDBJ Sequence Read Archive with accession number DRA007106.
