# Supplementary material for: Integrated transcriptomic and proteomic analyses of a molecular mechanism of radular teeth biomineralization in Cryptochiton stelleri
Source: Sci Rep. 2019 Jan 29;9:856. doi: 10.1038/s41598-018-37839-2 (PMC6351634; doi:10.1038/s41598-018-37839-2)
Supplement: Supplementary file 1 — Supplementary Information [file 41598_2018_37839_MOESM1_ESM.pdf]

Integrated transcriptomic and proteomic analyses of a molecular mechanism of radular teeth biomineralization in *Cryptochiton stelleri*

Michiko Nemoto, Dongni Ren, Steven Herrera, Songqin Pan, Takashi Tamura, Kenji Inagaki and David Kisailus

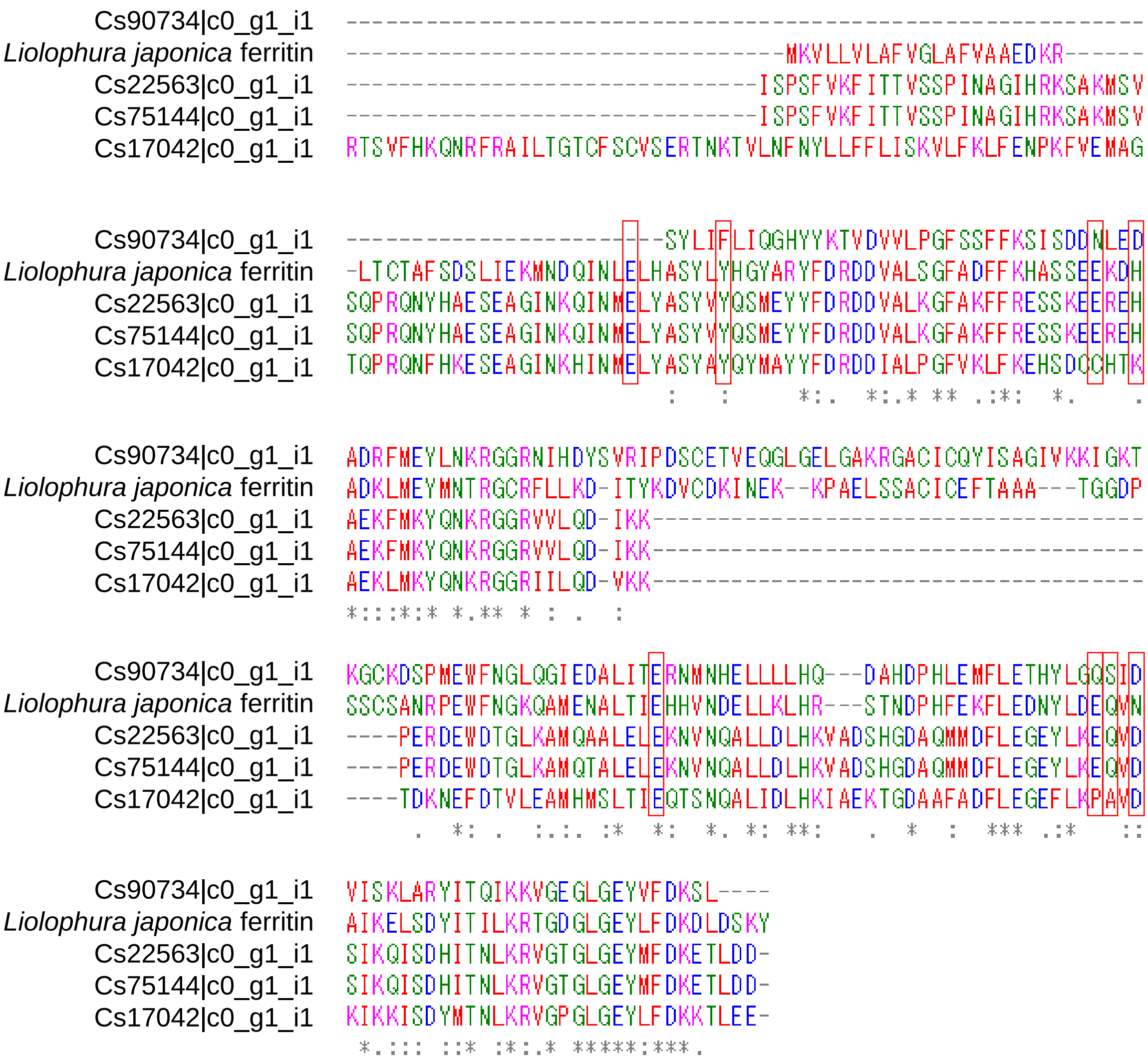

Figure S1 Multiple alignment of ferritins from *C. stelleri* and *L. japonica*. Amino acid sequences of *C. stelleri* ferritins were deduced from cDNA sequences. Conserved iron binding sites are boxed. Cs17042|c0_g1_i1 lacked the 5’ terminus of the cDNA.

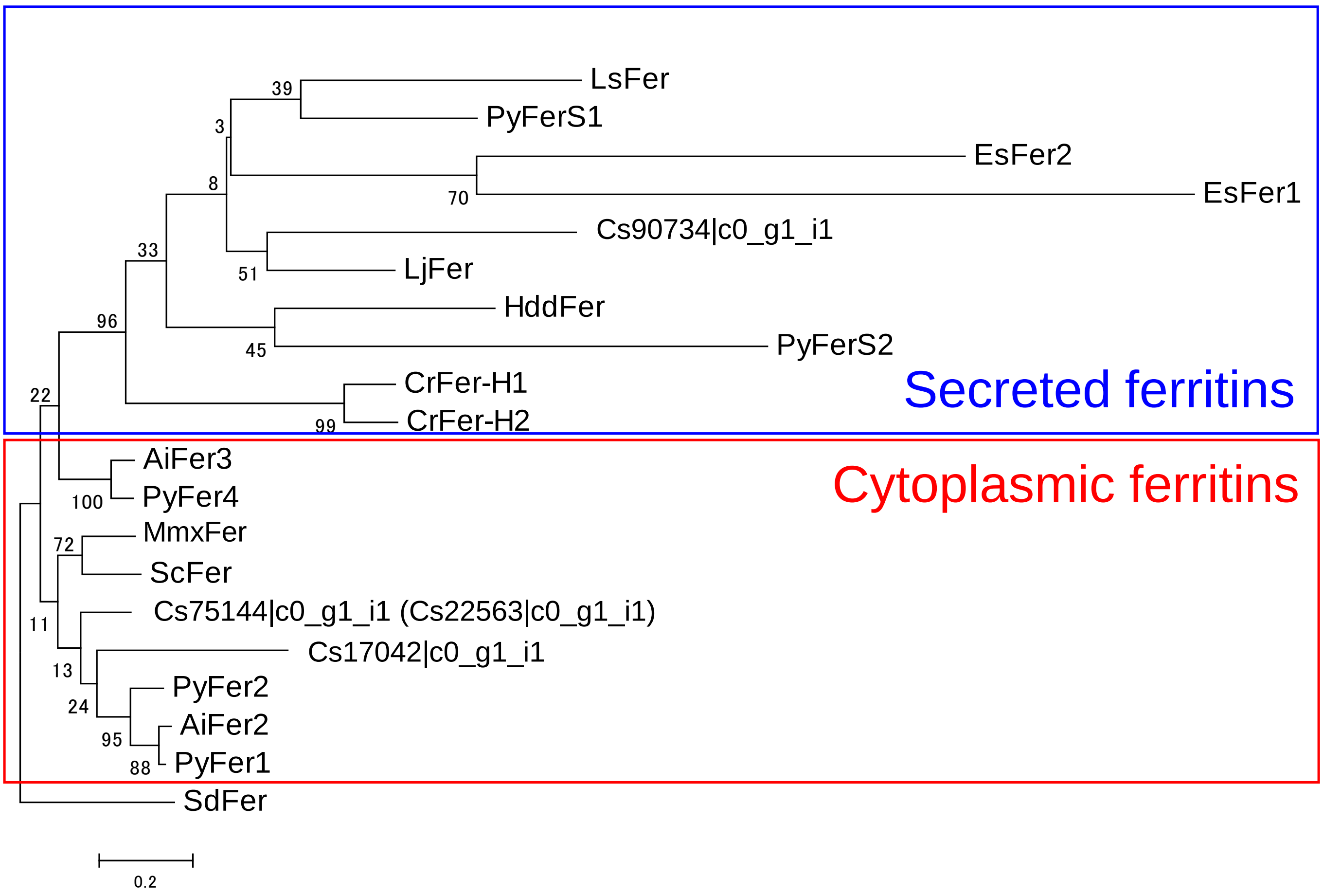

Figure S2 Phylogenetic tree of *C. stelleri* ferritins with other ferritins from Mollusca and Arthropoda.

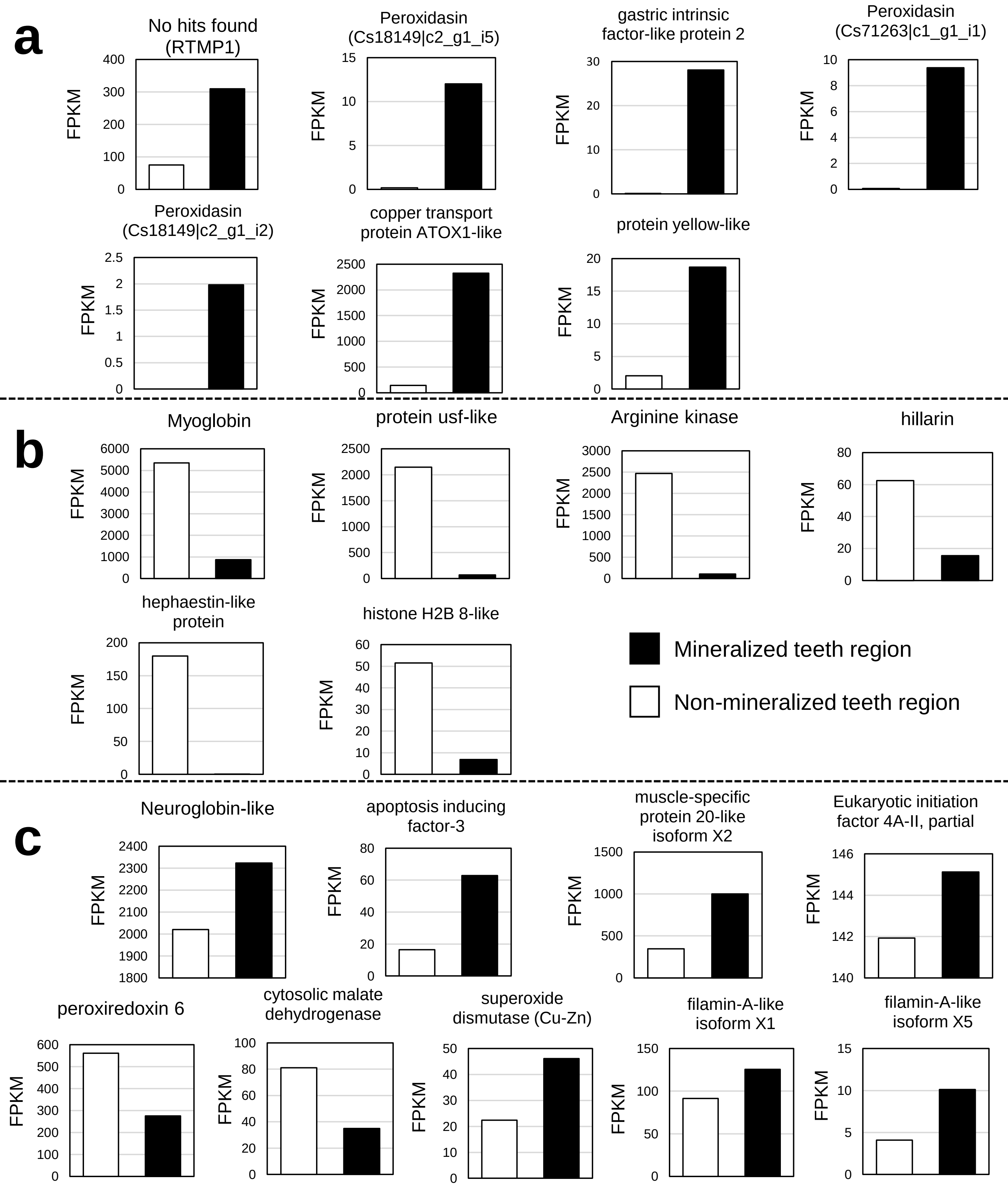

Figure S3 Expression of the transcripts encoding the mineralized cusp-specific proteins. (a) The transcripts that increased more than 4-fold at mineralized teeth region, (b) The transcripts that increased more than 4-fold at non-mineralized teeth region, (c) The transcripts differed in abundance by less than 4-fold between two regions.

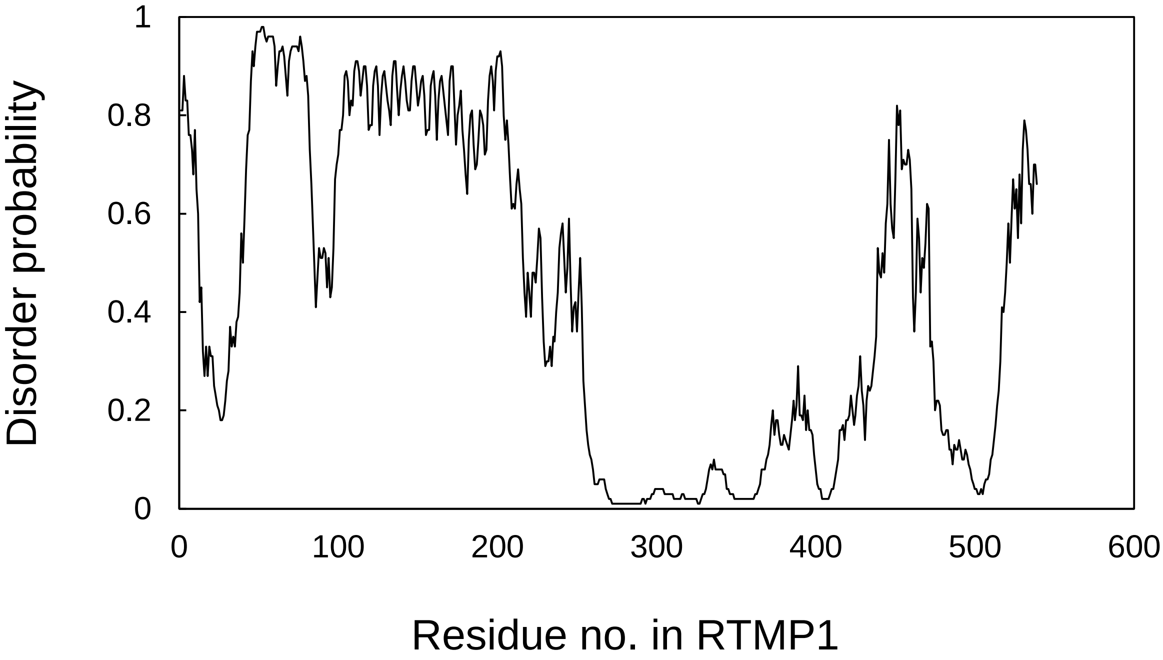

Figure S4 Disorder regions in RTMP1 were predicted with DISOPRED3[70].

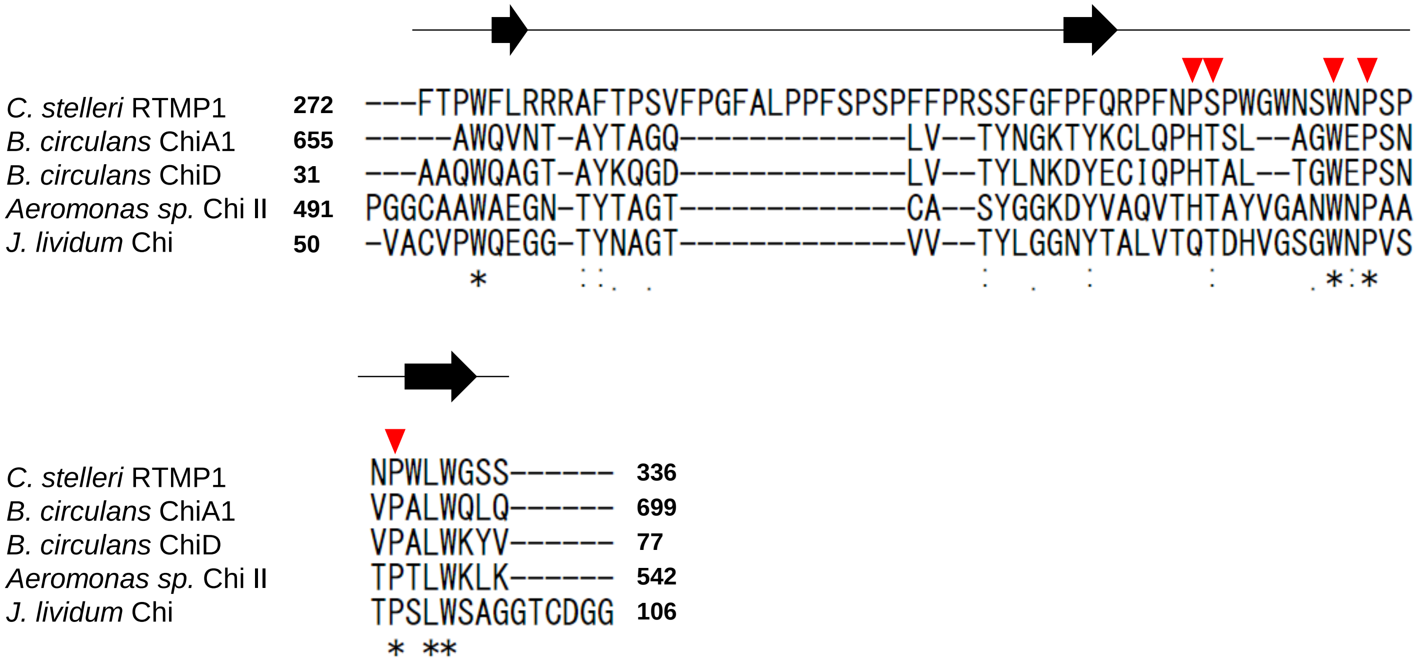

Figure S5 Amino acid sequence alignment of the WF-rich region of RTMP1 with the domains of other chitin binding proteins. The amino acid sequences shown are for *C. stelleri* RTMP1, *Bacillus circulans* WL-12 chitinase A1, *B. circulans* WL-12 chitinase D, *Aeromonas* sp. strain 10S-24 chitinase II and *Janthinobacterium lividum* chitinase. The black arrows at the top represent the predicted β-strand regions of RTMP1. The residues of the chitin binding proteins proposed to be candidates for the interaction with chitin are indicated by red arrow heads[31].

Table S1. List of the twenty most highly expressed transcripts in non-mineralized teeth region

Transcript ID	Annotation[a]	Best hit organisms	E-value	Function
Cs79475|c0_g1_i1	peritrophin-1-like	*Lottia gigantea*	6.00E-76	Unknown
Cs17717|c0_g1_i5	No hits found			Unknown
Cs17717|c0_g1_i3	No hits found			Unknown
Cs17717|c0_g2_i1	No hits found			Unknown
Cs37223|c0_g1_i1	No hits found			Unknown
Cs70642|c0_g2_i1	PREDICTED: peritrophin-44-like	*Octopus bimaculoides*	4.00E-111	peritrophic membrane protein, chitin-binding
Cs75144|c0_g1_i1	ferritin subunit 2	*Hyriopsis schlegelii*	1.00E-102	Iron storage
Cs17717|c0_g1_i4	No hits found			Unknown
Cs17717|c0_g1_i1	No hits found			Unknown
Cs62747|c0_g1_i1	PREDICTED: cell migration-inducing and hyaluronan-binding protein isoform X2	*Erinaceus europaeus*	3.00E-180	Regulation of cell migration
Cs77196|c0_g2_i1	Myoglobin	*Liolophura japonica*	4.00E-79	Oxygen transport
Cs87163|c0_g1_i1	No hits found			Unknown
Cs47470|c2_g3_i1	Actin	*Aplysia californica*	0	Cytoskeletal protein
Cs37223|c0_g2_i1	No hits found			Unknown
Cs17717|c0_g1_i2	No hits found			Unknown
Cs76629|c0_g1_i1	cytochrome c oxidase subunit I (mitochondrion)	*Cryptochiton stelleri*	0	Energy metabolism.
Cs24354|c0_g1_i1	elongation factor 1 alpha	*Axinella verrucosa*	0	transcription
Cs56877|c0_g1_i1	Platelet glycoprotein Ib alpha chain, partial	*Stegodyphus mimosarum*	2.00E-14	surface membrane protein of platelets
Cs25220|c1_g1_i1	PREDICTED: protein PIF-like	*Octopus bimaculoides*	2.00E-85	Aragonite biomineralization, chitin-binding
Cs82664|c0_g1_i1	Arginine kinase	*Liolophura japonica*	0	phosphotransferase

[a] Annotation is based on the results of BlastX analysis

Table S2. List of the twenty most highly expressed transcripts in mineralized teeth region

Transcript ID	Annotation[a]	Best hit organisms	E-value	Function
Cs58803|c0_g1_i1	hypothetical protein OXYTRI_13058 (macronuclear)	*Oxytricha trifallax*	1.00E-167	Unknown
Cs76629|c0_g1_i1	cytochrome c oxidase subunit I (mitochondrion)	*Cryptochiton stelleri*	0	Energy metabolism.
Cs43066|c0_g1_i1	cytochrome c oxidase subunit II (mitochondrion)	*Cryptochiton stelleri*	5.00E-149	Energy metabolism.
Cs26640|c0_g1_i1	cytochrome c oxidase subunit III (mitochondrion)	*Cryptochiton stelleri*	8.00E-148	Energy metabolism.
Cs43066|c1_g2_i1	ATP synthase F0 subunit 6 (mitochondrion)	*Cryptochiton stelleri*	2.00E-116	Energy metabolism.
Cs22243|c0_g6_i1	No hits found			Unknown
Cs22243|c0_g7_i3	No hits found			Unknown
Cs22243|c0_g7_i5	No hits found			Unknown
Cs22243|c0_g7_i6	No hits found			Unknown
Cs22243|c0_g7_i4	No hits found			Unknown
Cs45977|c0_g3_i1	hypothetical protein X975_22095, partial	*Stegodyphus mimosarum*	3.00E-19	Unknown
Cs45977|c0_g2_i1	hypothetical protein X975_22095, partial	*Stegodyphus mimosarum*	3.00E-18	Unknown
Cs22243|c0_g7_i2	No hits found			Unknown
Cs22243|c0_g5_i1	No hits found			Unknown
Cs71309|c7_g4_i1	antileukoproteinase-like	*Sorex araneus*	5.00E-09	Acid-stable proteinase inhibitor
Cs46263|c1_g1_i1	NADH dehydrogenase subunit 5 (mitochondrion)	*Cryptochiton stelleri*	0	Energy metabolism.
Cs50074|c0_g1_i1	NADH dehydrogenase subunit 1 (mitochondrion)	*Cryptochiton stelleri*	0	Energy metabolism.
Cs12250|c0_g1_i1	chitin binding beak protein 3	*Dosidicus gigas*	1.00E-09	defines the squid beak mechanical gradient
Cs46263|c0_g1_i1	cytochrome b (mitochondrion)	*Cryptochiton stelleri*	0	Energy metabolism.
Cs45977|c0_g1_i1	hypothetical protein OCBIM_22005804mg	*Octopus bimaculoides*	2.00E-25	Unknown

[a] Annotation is based on the results of BlastX analysis

Table S3. Expression profiles of ferritin encoding transcripts in *C. stelleri*

**Transcript ID**	**FPKM**	
	**Non-mineralized**	**Mineralized**
Cs22563|c0_g1_i1	113.11	3.54
Cs75144|c0_g1_i1	8240.36	238.32
Cs17042|c0_g1_i1	15.29	0.71
Cs90734|c0_g1_i1	1.17	0.8

Table S4. List of the proteins identified from mineralized cusp fraction of radular teeth in *Cryptochiton stelleri*

Annotation[a]	Best hit organisms	E-value	1st analysis		2nd analysis		Transcript ID	FPKM (mineralized)	FPKM (non-mineralized)
			Mascot scores	Number of peptides	Mascot scores	Number of peptides			
Neuroglobin-like	*Actinia tenebrosa*	2.00E-25	845	4	428	4	Cs77024|c0_g1_i1	2323.65	2020.22
							Cs52177|c6_g1_i1	68.63	41.13
actin	*Modiolus modiolus*	1.00E-86	485	4			Cs75591|c0_g1_i1	2.78	0.29
Actin	*Aplysia californica*	0	529	6	237	4	Cs47470|c2_g3_i1	2810	5142.67
							Cs47470|c1_g1_i1	54.41	68.29
ependymin-related protein 1-like	*Lottia gigantea*	1.00E-33	525	6	345	5	Cs63606|c4_g2_i1	15.21	2.52
							Cs44691|c0_g1_i1	605.89	110.22
inter-alpha-trypsin inhibitor heavy chain H3-like isoform X2	*Lingula anatina*	3.00E-145	452	4	269	6	Cs47656|c0_g1_i2	17.7	2.27
							Cs47656|c0_g1_i1	112	2.24
filamin-A isoform X14	*Crassostrea gigas*	0	453	7	163	3	Cs22318|c0_g1_i1	66.57	125.82
tubulin beta chain	*Hydra vulgaris*	0	333	5	150	3	Cs54304|c1_g2_i5	101.68	647.37
							Cs71671|c4_g2_i1	1.33	0
							Cs71671|c4_g2_i3	0	1.4
							Cs71671|c4_g2_i4	0	1.49
							Cs71671|c4_g2_i5	0	7.36
Myoglobin	*Liolophura japonica*	3.00E-84	387	4	234	3	Cs77196|c0_g1_i1	75.14	140.63
							Cs78790|c0_g1_i1	29.05	124.08
							Cs77196|c0_g2_i1	874.48	5345.03
protein usf-like	*Octopus bimaculoides*	1.00E-101	331	5	54	1	Cs75674|c0_g4_i1	67.74	2144.29
							Cs81542|c0_g1_i1	0.78	45.96
alpha tubulin-like	*Lottia gigantea*	0			200	3	Cs88263|c0_g1_i1	189.83	1835.9
							Cs92361|c0_g1_i1	0	10.36
alpha tubulin-like	*Saccoglossus kowalevskii*	0	278	3	212	3	Cs88263|c0_g1_i2	603.6	1986.65
							Cs88263|c1_g1_i1	0.97	16.82
							Cs88263|c1_g1_i2	7.73	13.5
zonadhesin-like	*Parus major*	2.00E-26	147	2	139	3	Cs23761|c3_g1_i1	2473.16	14.36
							Cs19070|c4_g1_i2	39.29	1.14
fibrillin-2-like	*Lingula anatina*	0	250	4	150	3	Cs22298|c0_g1_i2	5.07	0.31
							Cs22298|c0_g1_i4	0.97	0.05
Arginine kinase	*Liolophura japonica*	0	189	4	178	2	Cs82664|c0_g1_i1	104.69	2469.32
							Cs79589|c0_g2_i1	0	16.41
							Cs79589|c0_g1_i1	1.66	22.46
CD109 antigen-like isoform X1	*Crassostrea gigas*	0	160	3			Cs41242|c0_g1_i2	333.61	2.37
							Cs41242|c0_g1_i1	22.1	0
Peroxidasin	*Crassostrea gigas*	0	173	3	195	3	Cs18149|c2_g1_i3	4.81	0
							Cs18149|c2_g1_i5	12.02	0.18
CD109 antigen-like isoform X2	*Crassostrea gigas*	0	163	3	398	9	Cs78436|c2_g1_i1	110.87	0.26
protein PIF-like	*Lottia gigantea*	1.00E-35			105	1	Cs55300|c0_g1_i1	0.3	0
							Cs55300|c0_g1_i2	1.94	0

<table>
<tr><td rowspan="2">tubulin beta-4B chain-like isoform X1</td><td rowspan="2">Acanthaster planci</td><td rowspan="2">0.00E+00</td><td rowspan="2">179</td><td rowspan="2">2</td><td rowspan="2">160</td><td rowspan="2">3</td><td>Cs71671|c1_g3_i1</td><td>2.59</td><td>4.37</td></tr>
<tr><td>Cs71671|c1_g2_i1</td><td>54.54</td><td>211.44</td></tr>
<tr><td>inter-alpha-trypsin inhibitor heavy chain H4, partial</td><td>Acanthisitta chloris</td><td>1.00E-09</td><td>127</td><td>2</td><td>98</td><td>2</td><td>Cs48764|c0_g1_i1</td><td>1.01</td><td>0.26</td></tr>
<tr><td rowspan="3">uncharacterized protein LOC111636702</td><td rowspan="3">Centruroides sculpturatus</td><td rowspan="3">4.00E-05</td><td rowspan="3">138</td><td rowspan="3">2</td><td rowspan="3">86</td><td rowspan="3">1</td><td>Cs17650|c1_g2_i1</td><td>8.73</td><td>0</td></tr>
<tr><td>Cs34146|c1_g1_i1</td><td>495.85</td><td>0.02</td></tr>
<tr><td>Cs34146|c1_g1_i2</td><td>436.38</td><td>0.02</td></tr>
<tr><td rowspan="2">No hits found</td><td rowspan="2"></td><td rowspan="2"></td><td rowspan="2">93</td><td rowspan="2">1</td><td rowspan="2">233</td><td rowspan="2">4</td><td>Cs75160|c0_g1_i1</td><td>11.77</td><td>0</td></tr>
<tr><td>Cs80392|c0_g1_i1</td><td>786.93</td><td>0</td></tr>
<tr><td>protein PIF isoform X2</td><td>Crassostrea gigas</td><td>1.00E-46</td><td></td><td></td><td>70</td><td>1</td><td>Cs55300|c1_g1_i1</td><td>1.38</td><td>0</td></tr>
<tr><td rowspan="4">No hits found</td><td rowspan="4"></td><td rowspan="4"></td><td rowspan="4">126</td><td rowspan="4">2</td><td rowspan="4">88</td><td rowspan="4">1</td><td>Cs68435|c0_g1_i2</td><td>56.26</td><td>316.81</td></tr>
<tr><td>Cs68435|c0_g1_i1</td><td>309.95</td><td>75</td></tr>
<tr><td>Cs11532|c0_g1_i1</td><td>1.11</td><td>1.95</td></tr>
<tr><td>Cs93074|c3_g1_i1</td><td>5.43</td><td>4.9</td></tr>
<tr><td rowspan="3">lysosomal aspartic protease isoform X1</td><td rowspan="3">Crassostrea gigas</td><td rowspan="3">0</td><td rowspan="3">122</td><td rowspan="3">2</td><td rowspan="3"></td><td rowspan="3"></td><td>Cs54082|c4_g3_i5</td><td>70.7</td><td>40.45</td></tr>
<tr><td>Cs64530|c0_g1_i1</td><td>1.22</td><td>0</td></tr>
<tr><td>Cs64530|c0_g1_i3</td><td>0.81</td><td>0.59</td></tr>
<tr><td>protein yellow-like</td><td>Lingula anatina</td><td>3.00E-103</td><td>109</td><td>1</td><td>90</td><td>1</td><td>Cs83384|c0_g1_i1</td><td>18.69</td><td>2.05</td></tr>
<tr><td rowspan="3">melanotransferrin-like</td><td rowspan="3">Aplysia californica</td><td rowspan="3">8.00E-28</td><td rowspan="3">118</td><td rowspan="3">1</td><td rowspan="3"></td><td rowspan="3"></td><td>Cs33876|c0_g1_i1</td><td>55.79</td><td>46.56</td></tr>
<tr><td>Cs33876|c0_g1_i2</td><td>104.35</td><td>35.82</td></tr>
<tr><td>Cs35685|c0_g1_i1</td><td>4.7</td><td>1.26</td></tr>
<tr><td rowspan="3">cell migration-inducing and hyaluronan-binding protein-like</td><td rowspan="3">Branchiostoma belcheri</td><td rowspan="3">3.00E-125</td><td rowspan="3">116</td><td rowspan="3">1</td><td rowspan="3"></td><td rowspan="3"></td><td>Cs72782|c0_g1_i1</td><td>0.08</td><td>402.97</td></tr>
<tr><td>Cs68459|c0_g1_i2</td><td>0.02</td><td>2.33</td></tr>
<tr><td>Cs68459|c0_g1_i1</td><td>0</td><td>3.7</td></tr>
<tr><td>Peroxidasin</td><td>Mizuhopecten yessoensis</td><td>1.00E-19</td><td>102</td><td>2</td><td>111</td><td>3</td><td>Cs18149|c2_g1_i2</td><td>1.98</td><td>0</td></tr>
<tr><td rowspan="2">hypothetical protein LOTGIDRAFT_236297</td><td rowspan="2">Lottia gigantea</td><td rowspan="2">3.00E-40</td><td rowspan="2">72</td><td rowspan="2">1</td><td rowspan="2">67</td><td rowspan="2">1</td><td>Cs88032|c0_g1_i1</td><td>38.08</td><td>0.29</td></tr>
<tr><td>Cs88032|c0_g1_i2</td><td>23.79</td><td>0.1</td></tr>
<tr><td>gastric intrinsic factor-like protein 2</td><td>Hyriopsis cumingii</td><td>2.00E-35</td><td>114</td><td>1</td><td>56</td><td>1</td><td>Cs62719|c1_g8_i1</td><td>28.05</td><td>0.08</td></tr>
<tr><td rowspan="10">apotosis inducing factor-3</td><td rowspan="10">Haliotis discus discus</td><td rowspan="10">0.00E+00</td><td rowspan="10">95</td><td rowspan="10">1</td><td rowspan="10">98</td><td rowspan="10">2</td><td>Cs58153|c0_g2_i1</td><td>43.99</td><td>19.63</td></tr>
<tr><td>Cs24699|c0_g2_i2</td><td>2.4</td><td>0</td></tr>
<tr><td>Cs24699|c0_g2_i1</td><td>4.73</td><td>1.61</td></tr>
<tr><td>Cs58153|c0_g2_i2</td><td>61.69</td><td>0</td></tr>
<tr><td>Cs58153|c0_g2_i3</td><td>1.11</td><td>0</td></tr>
<tr><td>Cs58153|c0_g2_i4</td><td>0</td><td>18.88</td></tr>
<tr><td>Cs58153|c0_g2_i5</td><td>62.87</td><td>16.52</td></tr>
<tr><td>Cs58153|c0_g2_i6</td><td>0</td><td>11.62</td></tr>
<tr><td>Cs58153|c0_g2_i7</td><td>0</td><td>0</td></tr>
<tr><td>Cs58153|c0_g2_i8</td><td>0</td><td>0</td></tr>
<tr><td rowspan="2">copper transport protein ATOX1-like</td><td rowspan="2">Exaiptasia pallida</td><td rowspan="2">3.00E-27</td><td rowspan="2">109</td><td rowspan="2">1</td><td rowspan="2">104</td><td rowspan="2">1</td><td>Cs22616|c0_g1_i1</td><td>2324.41</td><td>144.53</td></tr>
<tr><td>Cs78473|c8_g1_i1</td><td>61.37</td><td>2.87</td></tr>
<tr><td rowspan="3">hillarin</td><td rowspan="3">Crassostrea virginica</td><td rowspan="3">0</td><td rowspan="3">83</td><td rowspan="3">1</td><td rowspan="3">61</td><td rowspan="3">1</td><td>Cs28725|c1_g1_i1</td><td>15.52</td><td>62.53</td></tr>
<tr><td>Cs28725|c1_g1_i2</td><td>17.63</td><td>33.26</td></tr>
<tr><td>Cs30794|c0_g1_i1</td><td>0</td><td>1.95</td></tr>
</table>

14-3-3 protein epsilon	*Dendroctonus ponderosae*	2.00E-148			101	1	Cs49664|c0_g1_i1	2.48	2.2
							Cs49664|c0_g2_i1	0	0
							Cs54082|c5_g1_i1	93.23	152.62
chitin binding beak protein 3	*Dosidicus gigas*	5.00E-10			52	1	Cs12250|c0_g1_i1	3170.58	0.28
							Cs71549|c0_g1_i1	87.72	0
muscle-specific protein 20-like isoform X2	*Lingula anatina*	7.00E-91	97	1	74	1	Cs50425|c0_g1_i2	17.41	6.06
							Cs84529|c0_g2_i1	999.04	345.27
Eukaryotic initiation factor 4A-II, partial	*Stegodyphus mimosarum*	0	95	1	85	1	Cs18720|c0_g1_i1	3.86	3.55
							Cs59400|c0_g1_i1	145.13	141.93
histone H2B 8-like	*Protobothrops mucrosquamatus*	2.00E-68	91	1	127	1	Cs55212|c0_g1_i2	4.08	0
							Cs55212|c0_g1_i1	1.61	0.49
							Cs74134|c0_g2_i1	0.52	15.62
							Cs74134|c0_g2_i2	6.85	51.47
							Cs74134|c0_g2_i4	0.31	9.28
							Cs74134|c0_g2_i5	3.21	2.81
							Cs74134|c0_g2_i6	7.35	3.45
							Cs74134|c0_g3_i1	2.27	2.79
lysosomal alpha-mannosidase	*Crassostrea gigas*	0			82	1	Cs17642|c2_g1_i1	42.49	36.06
							Cs26650|c0_g1_i2	0.28	0.4
neurexin-4-like	*Mizuhopecten yessoensis*	0			81	1	Cs29485|c0_g1_i1	37.55	40.55
							Cs43558|c0_g1_i1	1.23	0.42
ubiquitin family protein	*Oikopleura dioica*	2.00E-128			79	1	Cs59998|c2_g1_i3	9.08	11.73
							Cs16655|c0_g1_i1	30.64	19.34
							Cs39013|c0_g1_i1	0	4.67
							Cs39013|c0_g2_i1	0.85	0.92
							Cs39013|c0_g3_i1	2.96	4.43
							Cs84216|c0_g1_i3	19.88	21.84
							Cs84800|c0_g1_i1	38.16	25
							Cs91127|c0_g1_i1	397.69	212.55
							Cs91127|c0_g2_i1	0	252.71
							Cs91127|c0_g3_i1	561.52	318.04
							Cs59998|c1_g1_i2	288.81	330.36
							Cs59998|c1_g1_i4	1234.58	652.14
							Cs84216|c0_g1_i2	2.17	1.83
							Cs59998|c1_g1_i1	2018.71	2111.01
							Cs84216|c0_g1_i1	53.85	152.12
							Cs59998|c1_g1_i3	248.35	304.91
							Cs59998|c2_g1_i2	13.27	8.81
							Cs59998|c2_g1_i5	2.34	2.23
							Cs59998|c2_g1_i1	23.16	9.17
							Cs59998|c2_g1_i4	0.55	0
							Cs13896|c0_g1_i1	705.54	508.63

hypothetical protein LOTGIDRAFT_171050	*Lottia gigantea*	2.00E-20	90	1			Cs27910|c6_g1_i2	3.71	1.05
							Cs85075|c0_g1_i1	289.36	45.69
							Cs85075|c0_g1_i2	304.44	118.11
							Cs85075|c0_g1_i3	122.72	14.49
							Cs27910|c6_g1_i1	10.98	1.82
							Cs85075|c0_g1_i4	105.51	15.21
hephaestin-like protein	*Stylophora pistillata*	0	90	1	84	2	Cs46312|c2_g1_i1	0.27	179.74
							Cs77488|c0_g2_i1	0	3.47
EGF-like domain-containing protein 2 isoform X3	*Octopus bimaculoides*	1.00E-25	89	1			Cs28544|c0_g2_i1	17.45	1.57
							Cs79867|c0_g1_i1	1.4	0.41
elongation factor 1 alpha	*Axinella verrucosa*	0	73	2	55	1	Cs24354|c0_g1_i1	1819.62	3625.82
							Cs83769|c0_g1_i1	29.02	47.26
cathepsin Z-like	*Mizuhopecten yessoensis*	2.00E-151	85	1			Cs50066|c0_g1_i1	130.55	55.51
							Cs38675|c0_g1_i1	2.76	1.09
gelsolin-like protein 2	*Acanthaster planci*	1.00E-155	84	1			Cs37973|c0_g2_i1	1.21	12.73
							Cs45844|c0_g1_i1	107.29	356.86
							Cs45844|c0_g1_i2	0	114.49
							Cs75047|c0_g2_i1	17.44	10.56
							Cs78032|c0_g1_i1	2.1	1.45
							Cs75047|c0_g1_i1	35	11.06
							Cs75047|c0_g1_i2	19.62	0
							Cs75047|c0_g1_i3	9.22	25.47
filamin-A-like isoform X1	*Biomphalaria glabrata*	0	75	1	147	2	Cs54305|c0_g1_i2	125.51	91.37
							Cs54305|c0_g1_i1	5.54	9.2
							Cs86837|c0_g1_i1	2.92	1.5
radixin isoform X3	*Crassostrea gigas*	0	63	1			Cs19220|c1_g1_i1	162.74	221.31
							Cs68499|c0_g2_i1	3.74	4.87
filamin-A-like isoform X3	*Crassostrea virginica*	0.00E+00	78	1			Cs53984|c1_g2_i4	0	0
							Cs26775|c0_g1_i1	0.4	1.24
							Cs53984|c1_g2_i10	0	8.84
							Cs53984|c1_g2_i11	7.35	0
							Cs53984|c1_g2_i3	4.73	3.48
							Cs53984|c1_g2_i7	3.27	3.2
							Cs53984|c1_g2_i9	0.82	12.38
peroxiredoxin 6	*Cristaria plicata*	4.00E-119	77	2	93	2	Cs59318|c0_g1_i1	274.95	561.11
							Cs83444|c0_g1_i1	3.9	5.94
Peroxidasin	*Crassostrea gigas*	0	55	1	70	1	Cs71263|c1_g1_i1	9.37	0.08
							Cs71263|c1_g1_i8	6.11	0.07
galaxin-like	*Acropora digitifera*	1.00E-12	71	1			Cs12196|c0_g1_i1	107.08	0.07
							Cs13013|c0_g1_i1	4.34	0
							Cs76396|c0_g2_i1	9.68	43.42
							Cs73246|c0_g2_i1	0	0.81

thioredoxin peroxidase	*Artemia franciscana*	1.00E-116	70	1			Cs73246|c0_g3_i1	0.33	0.38
							Cs73246|c0_g4_i1	0.47	0
							Cs76396|c0_g1_i1	3.63	41.8
							Cs73246|c0_g1_i1	0.5	1.06
							Cs76396|c0_g3_i1	10.78	3.51
							Cs76396|c0_g4_i1	17	31.17
							Cs76396|c0_g5_i1	7.64	16.82
superoxide dismutase (Cu-Zn)	*Drosophila sechellia*	6.00E-46	76	1	190	2	Cs69741|c0_g1_i1	46.09	22.35
retrograde protein of 51 kDa-like isoform X4	*Crassostrea virginica*	3.00E-136	70	1	70	1	Cs20050|c0_g2_i1	33.69	1.12
							Cs34327|c0_g1_i1	1683.79	60.17
peptidyl-prolyl cis-trans isomerase-like	*Stylophora pistillata*	1.00E-89	57	1			Cs67145|c0_g1_i1	319.14	362.41
							Cs30224|c0_g1_i1	6.07	4.7
filamin-A-like isoform X5	*Crassostrea virginica*	2.00E-104	69	1	132	2	Cs53984|c1_g2_i1	4.69	3.34
							Cs53984|c1_g2_i6	6.78	5.64
							Cs53984|c1_g2_i8	10.13	4.09
CKLF-like MARVEL transmembrane domain-containing protein 4	*Lingula anatina*	8.00E-38			97	1	Cs93043|c0_g1_i1	9.57	0
							Cs93043|c0_g1_i2	6.74	5.05
Profilin	*Tuber melanosporum Mel28*	3.00E-10			67	1	Cs35744|c0_g2_i1	4.1	4.57
							Cs35744|c0_g1_i1	1.07	0
							Cs93141|c42_g7_i1	168.38	243.11
triosephosphate isomerase-like	*Branchiostoma belcheri*	1.00E-133			66	1	Cs62667|c1_g1_i1	24.59	63.64
							Cs41497|c0_g2_i1	0.16	0.72
							Cs41497|c0_g3_i1	0.16	0
							Cs41497|c0_g1_i1	0.16	1.08
CD109 antigen-like	*Lottia gigantea*	0			64	1	Cs93025|c0_g1_i1	7.22	21.47
clathrin heavy chain 1 isoform X2	*Mizuhopecten yessoensis*	0	64	1			Cs59011|c0_g1_i2	21.69	33.63
							Cs59011|c0_g1_i1	16.73	24.6
ubiquitin, partial (macronuclear)	*Nyctotherus ovalis*	7.00E-72	61	1			Cs70580|c0_g2_i1	1.53	0
cytosolic malate dehydrogenase	*Mytilus galloprovincialis*	3.00E-176	61	1	59	1	Cs28687|c2_g1_i1	34.88	81.1
							Cs21624|c0_g1_i1	0.94	1.56
ADP-ribosylation factor 1-like isoform X3	*Saccoglossus kowalevskii*	2.00E-117			58	1	Cs22952|c1_g1_i1	2.86	3.71
							Cs87098|c0_g1_i1	125.7	195.08
14-3-3 protein zeta isoform X1	*Dinoponera quadriceps*	1.00E-130			53	1	Cs74650|c0_g1_i1	182.51	195.15
							Cs84401|c0_g1_i1	5.71	4.62
nucleolin-like	*Lingula anatina*	8.00E-31			52	1	Cs73306|c0_g1_i3	113.67	238.35
							Cs21433|c0_g1_i2	3.39	4.94
							Cs21433|c0_g1_i4	0.93	0.37
							Cs73306|c0_g1_i2	3.5	4.1
							Cs21433|c0_g1_i1	0	0.34
							Cs73306|c0_g1_i1	27.18	29.24
F-actin-capping protein subunit alpha-2-like	*Lingula anatina*	6.00E-169			52	1	Cs83170|c0_g1_i1	37.69	72.2
							Cs31098|c0_g1_i1	1.04	1.99

tyrosine-protein kinase receptor Tie-1-like	*Lingula anatina*	4.00E-09	57	1			Cs33897|c6_g1_i7	26.47	44.23
							Cs33897|c4_g1_i3	0.56	0
							Cs33897|c4_g1_i4	1.7	0
							Cs33897|c4_g1_i5	0	0.44
							Cs33897|c6_g1_i4	68.56	21.8
							Cs33897|c4_g1_i2	0.74	1.78
							Cs33897|c6_g1_i1	50.57	79
							Cs33897|c6_g1_i6	50.98	49.12
							Cs33897|c6_g1_i5	47.34	0
							Cs33897|c6_g1_i2	33.26	0
NADP-dependent malic enzyme	*Crassostrea gigas*	0	57	1			Cs92452|c0_g1_i1	2.44	8.79
dipeptidyl peptidase 1-like isoform X1	*Crassostrea virginica*	0.00E+00	55	1			Cs92769|c1_g1_i1	70.84	14.85
							Cs28054|c0_g1_i1	2.33	0.3
nucleoside diphosphate kinase A	*Crassostrea gigas*	1.00E-76	54	1			Cs27778|c0_g1_i1	427.75	93.45
							Cs27778|c0_g2_i1	0	650.63
							Cs67705|c0_g1_i1	0	11.49
							Cs67705|c0_g2_i1	9.01	0.85
Phosphoenolpyruvate carboxykinase [GTP]	*Lottia gigantea*	0.00E+00	53	1			Cs63416|c0_g1_i1	0.72	1.31
							Cs81194|c0_g1_i1	22.04	31.08
alpha-L-fucosidase-like	*Mizuhopecten yessoensis*	0	51	1			Cs82886|c4_g3_i2	2.95	30.29
							Cs82886|c4_g3_i4	4.73	0
collagen alpha-1(XII) chain-like	*Octopus bimaculoides*	5.00E-24	51	1			Cs24988|c0_g1_i3	1.83	0.12
							Cs24988|c0_g1_i4	0.42	0
							Cs24988|c0_g1_i7	0	0
							Cs24988|c0_g1_i8	10.12	1.23

[a] Annotation is based on the results of BlastP analysis

Table S5. List of the proteins identified from base plus membrane fraction of radular teeth in *Cryptochiton stelleri*

Annotation[a]	Best hit	E-value	1st analysis		2nd analysis		Transcript ID	FPKM (mineralized)	FPKM (non-mineralized)
			Mascot scores	number of peptides	Mascot scores	number of peptides			
inter-alpha-trypsin inhibitor heavy chain H3-like isoform X2	*Lingula anatina*	3.00E-145	239	3	81	2	Cs47656|c0_g1_i2	17.7	2.27
							Cs47656|c0_g1_i1	112	2.24
CD109 antigen-like isoform X2	*Crassostrea gigas*	0	233	5	490	10	Cs78436|c2_g1_i1	110.87	0.26
zonadhesin-like	*Parus major*	2.00E-26	233	3	165	2	Cs19070|c4_g1_i2	39.29	1.14
							Cs23761|c3_g1_i1	2473.16	14.36
alpha tubulin-like	*Lottia gigantea*	0			134	2	Cs81533|c0_g1_i1	1.48	1.05
							Cs81533|c0_g2_i1	0	1.22
							Cs88263|c0_g1_i1	189.83	1835.9
							Cs88263|c0_g1_i3	901.4	732.35
							Cs92361|c0_g1_i1	0	10.36
							Cs92361|c1_g3_i1	18.12	0.22
hypothetical protein	*Lottia gigantea*	2.00E-50	220	4	79	2	Cs40476|c0_g1_i1	6.45	92.83
							Cs83855|c0_g1_i1	0.13	2.38
Actin	*Aplysia californica*	0	216	3	99	3	Cs47470|c1_g1_i1	54.41	68.29
							Cs47470|c2_g3_i1	2810	5142.67
actin	*Modiolus modiolus*	1.00E-86	194	1			Cs75591|c0_g1_i1	2.78	0.29
CD109 antigen-like isoform X1	*Crassostrea gigas*	0	191	2	61	1	Cs41242|c0_g1_i1	22.1	0
							Cs41242|c0_g1_i2	333.61	2.37
							Cs70978|c0_g1_i1	21.54	0
							Cs84232|c0_g1_i1	11.94	0
retrograde protein of 51 kDa-like isoform X4	*Crassostrea virginica*	3.00E-136	137	2	178	3	Cs20050|c0_g2_i1	33.69	1.12
							Cs34327|c0_g1_i1	1683.79	60.17
No hits found			129	1	68	1	Cs32565|c1_g1_i1	40.95	0
							Cs32565|c1_g1_i2	5.45	0.67
malate dehydrogenase, mitochondrial isoform X2	*Aplysia californica*	0	121	1			Cs75179|c0_g1_i1	77.41	43.32
							Cs75179|c0_g1_i2	0	10.36
fibrillin-2-like	*Lingula anatina*	0	113	3	62	1	Cs22298|c0_g1_i2	5.07	0.31
							Cs22298|c0_g1_i4	0.97	0.05
uncharacterized protein LOC111636702	*Centruroides sculpturatus*	4.00E-05	105	2	263	3	Cs17650|c1_g2_i1	8.73	0
							Cs34146|c1_g1_i1	495.85	0.02
							Cs34146|c1_g1_i2	436.38	0.02
cell surface protein	*Corynebacterium phoceense*	2.00E-06	94	1			Cs58933|c0_g1_i1	2.91	0
							Cs65631|c0_g1_i1	27.59	1.17
peroxidasin-like	*Lottia gigantea*	1.00E-152	80	1			Cs81561|c0_g1_i1	1.51	0.35
							Cs11665|c0_g1_i1	6.87	74.38
							Cs49435|c0_g1_i1	3.19	193.6
							Cs54304|c1_g2_i1	110.53	2
							Cs54304|c1_g2_i2	53.84	69.25
							Cs54304|c1_g2_i3	6.3	104.23

tubulin beta chain	*Hydra vulgaris*	0	70	1	62	1	Cs54304|c1_g2_i4	0	0
							Cs54304|c1_g2_i5	101.68	647.37
							Cs54304|c1_g2_i6	2.43	19.67
							Cs54304|c1_g4_i1	0.16	36.87
							Cs71671|c1_g2_i1	54.54	211.44
							Cs71671|c1_g3_i1	2.59	4.37
							Cs71671|c1_g3_i2	76.04	1.78
							Cs71671|c1_g3_i3	0	0
							Cs71671|c1_g3_i4	14.97	10.72
							Cs71671|c4_g2_i1	1.33	0
							Cs71671|c4_g2_i3	0	1.4
							Cs71671|c4_g2_i8	0.74	0
							Cs71671|c4_g3_i1	1.05	3.49
							Cs71671|c4_g2_i2	3.3	4.2
							Cs71671|c4_g2_i4	0	1.49
							Cs71671|c4_g2_i5	0	7.36
							Cs71671|c4_g2_i9	0.68	0
No hits found			62	1			Cs43469|c0_g1_i1	2.01	0
							Cs85396|c0_g1_i1	151.1	0
collagen alpha-1(XII) chain-like	*Octopus bimaculoides*	9.00E-28	61	1			Cs24988|c0_g1_i1	2.58	0
							Cs24988|c0_g1_i11	0.62	0.15
							Cs24988|c0_g1_i5	12.64	6.02
							Cs24988|c0_g1_i6	0	0
uncharacterized protein LOC110454775	*Mizuhopecten yessoensis*	3.00E-24	57	1			Cs61402|c0_g1_i1	2	2.88
							Cs61402|c0_g1_i2	7.16	1.49
							Cs75949|c0_g1_i1	379.51	272.39
alpha tubulin-like	*Saccoglossus kowalevskii*	0	56	1	126	2	Cs20163|c0_g1_i1	111.79	165.29
							Cs36028|c0_g1_i1	0	17.03
							Cs36028|c0_g2_i1	0	0
							Cs36028|c0_g3_i1	0	0
							Cs43007|c0_g2_i1	0.44	30.38
							Cs43007|c0_g4_i1	16.57	2.92
							Cs64094|c0_g1_i1	0	10.52
							Cs64094|c0_g1_i2	4.73	34.2
							Cs72036|c0_g1_i1	1.42	1.72
							Cs88263|c0_g1_i1	189.83	1835.9
							Cs88263|c0_g1_i2	603.6	1986.65
							Cs88263|c1_g1_i1	0.97	16.82
							Cs88263|c1_g1_i2	7.73	13.5
							Cs88263|c1_g1_i3	6.88	11.91
							Cs88573|c0_g1_i1	0.66	3.55
							Cs92361|c0_g1_i1	0	10.36

							Cs92361|c0_g1_i2	0.76	1.88
No hits found			54	1			Cs78604|c1_g2_i2	20	0.68
chitin binding beak protein 3	*Dosidicus gigas*	5.00E-10	53	1	65	1	Cs12250|c0_g1_i1	3170.58	0.28
							Cs71549|c0_g1_i1	87.72	0
No hits found			52	1	51	1	Cs17717|c0_g1_i3	38.82	11750.12
							Cs17717|c0_g1_i4	105.68	7613.02
							Cs17717|c1_g1_i1	0.63	122.76
							Cs17717|c1_g1_i4	0.58	206.04
							Cs17717|c0_g1_i2	21	3943.71
							Cs17717|c0_g1_i1	41.73	6108.95
No hits found					122	2	Cs75160|c0_g1_i1	11.77	0
							Cs80392|c0_g1_i1	786.93	0
putative cysteine proteinase CG12163	*Branchiostoma belcheri*	9.00E-33			110	1	Cs85860|c0_g2_i1	4.05	0
uncharacterized protein LOC106869807	*Octopus bimaculoides*	6.00E-09			87	1	Cs35465|c0_g1_i1	73.35	3.38
							Cs62172|c0_g1_i1	2.54	0
tropomyosin-1 isoform X19	*Crassostrea virginica*	8.00E-65			68	1	Cs85863|c1_g1_i3	0	26.24
							Cs21476|c0_g1_i1	2.56	2.92
							Cs21476|c0_g1_i2	2.5	0.29
							Cs85863|c1_g1_i1	150.91	107.33
							Cs85863|c1_g1_i2	35.78	4.81
ependymin-related protein 1-like	*Lottia gigantea*	1.00E-33			64	1	Cs63606|c4_g1_i1	0	0
							Cs63606|c4_g2_i1	15.21	2.52
							Cs44691|c0_g1_i1	605.89	110.22
filamin-A isoform X14	*Crassostrea gigas*	0			62	1	Cs13902|c0_g1_i1	0.57	2.96
							Cs22318|c0_g1_i1	66.57	125.82
transcription factor with AP2 domain(s), putative	*Plasmodium malariae*	2.00E-04			60	1	Cs22247|c2_g1_i1	331.87	0.21
							Cs22247|c2_g1_i3	123	0
							Cs22247|c3_g1_i12	0	0
							Cs22247|c3_g1_i3	4.24	0
							Cs22247|c3_g1_i9	2.98	0
inter-alpha-trypsin inhibitor heavy chain H4, partial	*Acanthisitta chloris*	1.00E-09			56	1	Cs48764|c0_g1_i1	1.01	0.26
hypothetical protein LOTGIDRAFT_236297	*Lottia gigantea*	3.00E-40			54	1	Cs88032|c0_g1_i1	38.08	0.29
							Cs88032|c0_g1_i2	23.79	0.1

[a] Annotation is based on the results of BlastP analysis

Table S6. Localization analysis of mineralized cusp-specific proteins

Transcript ID	Annotation[a]	Signal sequence prediction software				
		**SignalP**	**TargetP[b]**	**PSORT**	**MultiLoc2**	**Signal-BLAST**
Cs77024|c0_g1_i1	Neuroglobin-like			cytosol	cytoplasmic	
Cs77196|c0_g2_i1	Myoglobin			cytosol	secretory pathway	
Cs75674|c0_g4_i1	protein usf-like			mitochondria	secretory pathway	○
Cs82664|c0_g1_i1	Arginine kinase		M	mitochondria	cytoplasmic	
Cs18149|c2_g1_i5	Peroxidasin	○	S	plasma membrnae	secretory pathway	○
Cs68435|c0_g1_i1	No hits found		S	extracellular	secretory pathway	○
Cs83384|c0_g1_i1	protein yellow-like	○	S	plasma membrnae	secretory pathway	○
Cs18149|c2_g1_i2[c]	Peroxidasin	○	S	plasma membrnae	secretory pathway	○
Cs62719|c1_g8_i1	gastric intrinsic factor-like protein 2	○	S	extracellular	secretory pathway	○
Cs58153|c0_g2_i5	apoptosis inducing factor-3			mitochondria	secretory pathway	
Cs22616|c0_g1_i1	copper transport protein ATOX1-like			extracellular	cytoplasmic	
Cs28725|c1_g1_i1	hillarin		M	mitochondria	cytoplasmic	
Cs84529|c0_g2_i1	muscle-specific protein 20-like isoform X2			cytosol	cytoplasmic	
Cs59400|c0_g1_i1	Eukaryotic initiation factor 4A-II, partial			nucleus	nuclear	
Cs74134|c0_g2_i2[c]	histone H2B 8-like			nucleus	nuclear	
Cs46312|c2_g1_i1	hephaestin-like protein		S	plasma membrnae	secretory pathway	○
Cs54305|c0_g1_i2[c]	filamin-A-like isoform X1			nucleus	cytoplasmic	
Cs59318|c0_g1_i1	peroxiredoxin 6			cytosol	cytoplasmic	
Cs71263|c1_g1_i1	Peroxidasin	○	S	extracellular	secretory pathway	
Cs69741|c0_g1_i1	superoxide dismutase (Cu-Zn)	○	S	extracellular	secretory pathway	○
Cs53984|c1_g2_i8	filamin-A-like isoform X5			cytosol	cytoplasmic	
Cs28687|c2_g1_i1	cytosolic malate dehydrogenase			mitochondria	cytoplasmic	

[a] Annotation is based on the results of BlastP analysis

[b] S, Secretory pathway; M, Mitochondrion

[c] cDNA of those proteins are likely truncated.
